# Supplementary material for: Molecular Classification of Genes Associated with Hypoxic Lipid Metabolism in Pancreatic Cancer
Source: Biomolecules. 2022 Oct 21;12(10):1533. doi: 10.3390/biom12101533 (PMC9599075; doi:10.3390/biom12101533)
Supplement: Supplementary file 1 [file biomolecules-12-01533-s001.zip › biomolecules-1941464-supplementary.pdf]

biomolecules

MDPI

Article

Molecular Classification of Genes Associated with Hypoxic Lipid Metabolism in Pancreatic Cancer

Supplementary

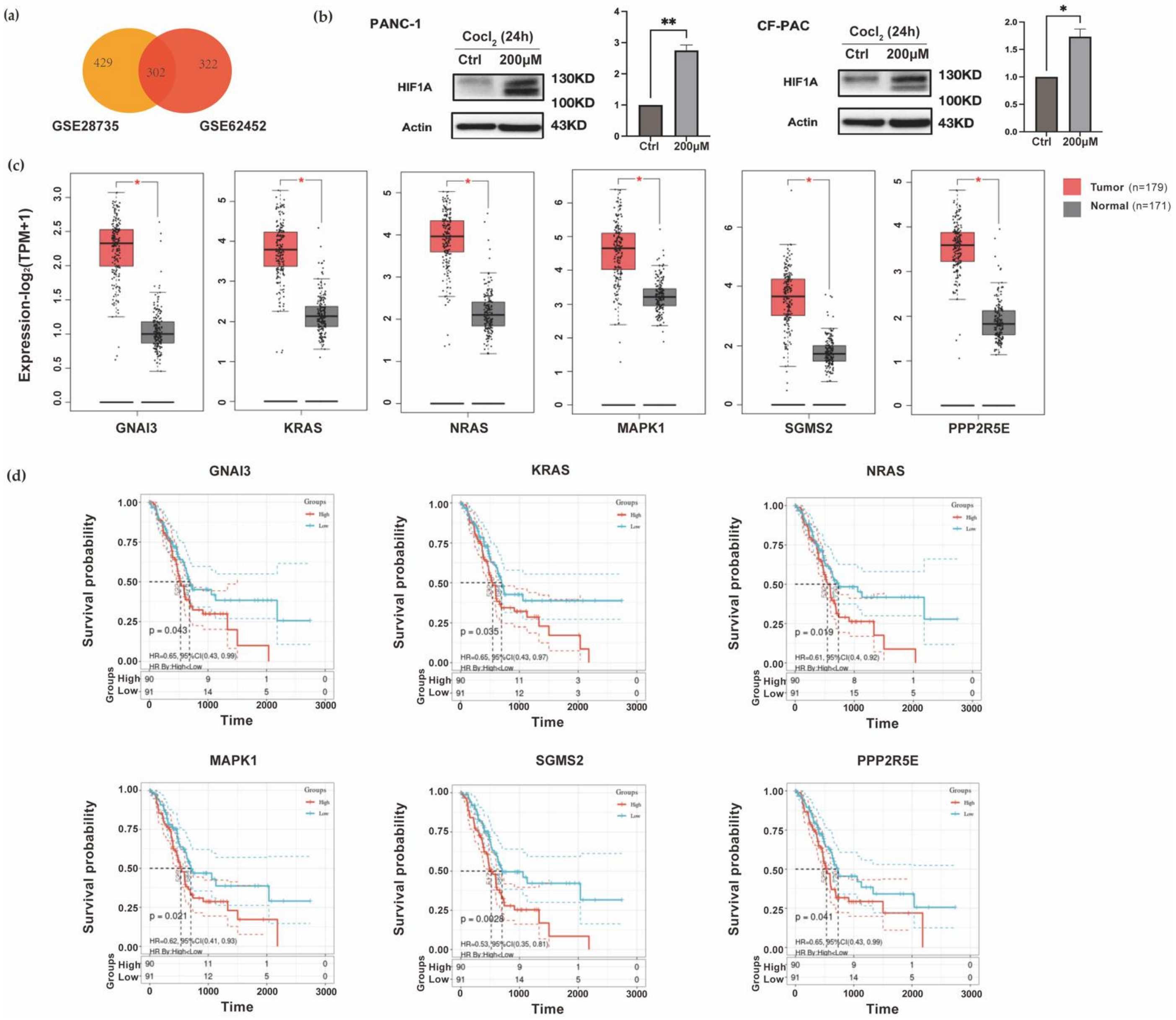

Figure S1. The hypoxia culture efficiency and supplement of data analysis in GEO and TCGA (a). Venn diagram with the number of genes analyzed in two GEO datasets (b). protein expression level of HIF-1α and actin of PDAC cells before and after hypoxia treatment. (n=3.*,p<0.05;**,p<0.01) (cropped)(The origion blots are in additional file-origion blot.pdf) (c). The mRNA expression level of six genes in TCGA and GTEx database. (*, p<0. 05) (d).Kaplan-Meier survival analysis of six genes in Sphingolipid signaling pathway. (GSE28735:https://www.ncbi.nlm.nih.gov/geo/query/acc.cgi?acc=gse28735; GSE62452:https://www.ncbi.nlm.nih.gov/geo/query/acc.cgi?acc=gse62452; TCGA:https://portal.gdc.cancer.gov/）.

Table S1. The annotation of metabolites shown in the heatmap of metabolic analysis.

Compound	CFPAC-1 (Cocl2/Ctrl)	PANC-1 (Cocl2/Ctrl)
METLIN_1-Palmitoyllysophosphatidylcholine_MID_182	3.308389596	0.007505441
METLIN_1,2-Dioleoyl_PC_MID_45202	359.5361017	1.12507489
METLIN_Bis(2-ethylhexyl)_phthalate_MID_263643	0.932044015	0.00927735
VGDB_L-Isoleucine_YID_00990	83.92849239	0.936690456
VGDB_N-Tetracosanoyl-4-sphingenyl-1-O-phosphorylcholine_YID_05090	14.13277004	0.17089418
VGDB_Ala-Gln_YID_00582	12.13238862	0.641294451
METLIN_N-Lignoceroylsphingosine_MID_63015	16.98559759	0.932033054
VGDB_2-Docosahexaenoyl-1-stearoyl-sn-glycero-3-phosphoserine_YID_11877	16.4339009	0.92179356
METLIN_Ceramide_(d18	13.94486939	0.807064334
VGDB_1,2-Di-(13Z-docosenoyl)-sn-glycero-3-phosphocholine_YID_11906	8.713625442	0.552735001
VGDB_1-Stearoyl-2-arachidonoyl-sn-glycero-3-phospho-(1'-myo-inositol)_YID_11903	13.10561572	0.843199098
VGDB_2-Oleoyl-1-palmitoyl-sn-glycero-3-phosphoserine_YID_11820	9.420347819	0.734978293
MASSBANK_S-Lactoylglutathione; LC-ESI-QTOF; MS2; CE	7.351777976	0.668413173
VGDB_Ala-Gln_YID_00582	6.255194808	0.573292073
METLIN_1,2-dipalmitoyl-sn-glycero-3-PC_MID_45182	9.886825432	0.994684981
METLIN_Adenosine_MID_86	3.972228694	0.435397105
VGDB_2-Docosahexaenoyl-1-stearoyl-sn-glycero-3-phosphoserine_YID_11877	6.072956626	0.79911369
MASSBANK_4-Pyridoxic acid; LC-ESI-QTOF; MS2; CE	1.116607564	0.158628687
MASSBANK_Inosine; LC-ESI-QTOF; MS2; CE	4.128480306	0.658655122
VGDB_2-Oleoyl-1-palmitoyl-sn-glycero-3-phosphocholine_YID_11817	6.839477889	1.107740633
VGDB_1-(1Z-Octadecenyl)-2-(4Z,7Z,10Z,13Z,16Z,19Z-docosahexaenoyl)-sn-glycero-3-phosphoethanolamine_YID_11841	5.138557482	0.847288021
VGDB_1-Stearoyl-2-oleoyl-sn-glycero-3-phosphoethanolamine_YID_06907	5.775859113	1.007205874
METLIN_Guanosine_MID_87	3.307238737	0.580276964
METLIN_N-Acetyl-D-glucosamine_MID_3356	4.603712003	0.815429767
VGDB_2-Hydroxy-6-aminopurine_YID_07007	3.322255029	0.636154012
MONA_Guanosine	3.392724156	0.671075439
METLIN_N-Acetyl-alpha-D-glucosamine_1-phosphate_MID_384	3.095448339	0.612486197
VGDB_Palmitoyl_sphingomyelin_YID_11776	4.587027818	0.912091202
VGDB_1,2-Dipalmitoyl-sn-glycero-3-phospho-(1'-rac-glycerol)_YID_11804	4.93397648	1.043539389
MONA_2-Thiocytidine	2.487400088	0.528617104
METLIN_Hypoxanthine_MID_83	3.676007321	0.794305414

VGDB_1-Myristoyl-2-palmitoyl-sn-glycero-3-phosphocholine_YID_11779	4.392732924	0.993469629
MASSBANK_Glucoraphanin; LC-ESI-ITFT; MS2; HCD; CE 55.0 eV; [M-H]-	14.3189005	3.331944338
METLIN_PG(16	4.391713908	1.027627122
METLIN_2',3'_cyclic_CMP_MID_62429	1.28552611	0.31512276
VGDB_1-Palmitoyl-3-oleoyl-sn-glycero-2-phos-phoethanolamine_YID_11795	4.106173075	1.013253197
METLIN_Asp_Phe_Lys_MID_18300	2.6214686	0.692160056
METLIN_2'-Deoxyadenosine_MID_3382	3.814154853	1.014706205
MONA_Glucoraphanin	12.6772156	3.389517081
METLIN_Vidarabine_MID_3020	2.232487382	0.597916668
METLIN_Inosine_MID_84	3.251564794	0.898834875
METLIN_Sucrose_MID_137	3.77333273	1.056825935
VGDB_1-Stearoyl-2-hydroxy-sn-glycero-3-phos-phocholine_YID_11215	4.476257849	1.275810366
MASSBANK_(-)-Nicotine; LC-ESI-QTOF; MS2; CE	1.530242735	0.463777697
MASSBANK_L-Glutamine; LC-ESI-QTOF; MS2; CE	1.098240469	0.341330244
VGDB_1,2-Dilinoleoyl-sn-glycero-3-phosphocho-line_YID_11846	3.22818618	1.081407251
VGDB_Robinin_YID_03141	5.817393173	1.97019593
MASSBANK_Phosphocholine; LC-ESI-QTOF; MS2; CE	3.465504279	1.174264666
MONA_Glucobrassicin	7.81094743	2.67497851
VGDB_Indole_YID_01039	2.082931237	0.740080036
METLIN_Allose_MID_348	2.28154659	0.814739294
PLASMA_Sinapic acid	9.558403881	3.487892794
VGDB_1-(9Z-Octadecenoyl)-sn-glycero-3-phos-phocholine_YID_06098	2.604411118	0.986608817
MASSBANK_Kaempferol-3-O-beta-D-galacto-side-7-O-alpha-L-rhamnoside; LC-ESI-QTOF; MS2; CE	3.6541576	1.438388049
MASSBANK_Sinapoyl malate; LC-ESI-QTOF; MS2; CE	8.893679188	3.531844778
VGDB_Triethanolamine_YID_02409	1.441865983	0.580350155
MASSBANK_Sarcinaxanthin monoglucoside; FAB-EBEB; MS2; m/z	3.033087245	1.244347844
VGDB_Arg-Asp_YID_05974	1.470869371	0.604665579
MASSBANK_Kaempferol-7-O-alpha-L-rhamno-side; LC-ESI-QTOF; MS2; CE	3.365975476	1.39004996
METLIN_Leucinic_acid_MID_64935	0.905883776	0.377003003
VGDB_1-Palmitoyl-2-linoleoyl-sn-glycero-3-phos-phocholine_YID_11815	2.703556759	1.151767419
VGDB_1-Octadecanoyl-sn-glycero-3-phosphoeth-anolamine_YID_06906	2.299019227	1.021576982
METLIN_Glycerophosphocholine_MID_370	0.987341233	0.459132217
VGDB_(-)-Riboflavin_YID_00817	1.677301164	0.809955805
METLIN_PS(16	1.588192507	0.77472941
METLIN_gamma-Glu-Cys_MID_3379	1.884642878	0.942906523

VGDB_1-Stearoyl-2-docosahexaenoyl-sn-glycero-3-phosphocholine_YID_11872	3.16636831	1.606799167
VGDB_2'-Hydroxy-3-methoxychalcone_YID_08452	1.606346514	0.841957659
METLIN_(3R,5R)-3,5-Dihydroxy-7-{(1S,2S,8S,8aR)-2-methyl-8-[(2-methylbutanoyl)oxy]-1,2,6,7,8,8a-hexahydro-1-naphthalenyl}heptanoic_acid_MID_985303	1.57995774	0.84776153
METLIN_PE(16	2.350514846	1.281098658
METLIN_Gamma-Glu-Leu_MID_23750	2.410776875	1.329079709
MASSBANK_Dicamba; LC-ESI-ITFT; MS2; CE	1.848558605	1.042206614
MASSBANK_Dehydro fucoxanthin acetate; FAB-EBEB; MS2; m/z	0.946592484	0.547950623
METLIN_Tyr_Glu_Gln_MID_22048	0.763663967	0.447365245
METLIN_Creatine_phosphate_MID_326	1.311322577	0.785516671
VGDB_.gamma.-Glu-Cys_YID_01201	1.707839467	1.051813492
Trimethylamine??N-oxide	1.206302774	0.74349403
METLIN_Cys_Ser_Gln_MID_21459	4.661339684	2.890899502
MASSBANK_GABA; LC-ESI-QTOF; MS2; CE	1.559360927	0.976447322
METLIN_L-a-Lysophosphatidylserine_MID_34531	1.809279866	1.150209731
METLIN_Hypotaurine_MID_281	1.221682676	0.819425906
METLIN_L-a-Lysophosphatidylserine_MID_34531	1.510851219	1.016015721
MASSBANK_1-O-beta-D-Glucopyranosyl sinapate; LC-ESI-QTOF; MS2; CE	2.355356821	1.585199671
VGDB_Threonic_acid_YID_11538	1.411715433	0.993854439
RESPECT_Choline	1.124415195	0.791955445
MASSBANK_Imidacloprid; LC-ESI-ITFT; MS2; CE	1.420583967	1.019042056
METLIN_Glycerophosphocholine_MID_370	1.370292282	0.993967115
METLIN_Thiamine_MID_229	1.047481233	0.766076383
METLIN_L-Tyrosine_MID_34	0.950501471	0.708338786
METLIN_PS(18	1.370405073	1.024152746
MASSBANK_4-Methyl-5-thiazoleethanol; LC-ESI-QTOF; MS2; CE	1.174107247	0.891207306
VGDB_Cytosine_YID_00961	1.62611736	1.237113917
VGDB_Anthranilic_acid_YID_00646	1.140193792	0.935121478
VGDB_N-Acetylglucosaminylasparagine_YID_02059	1.055947981	0.879469937
METLIN_N-nonanoyl-L-Homoserine_lactone_MID_45725	0.941495199	0.789501011
VGDB_bata-Hydroxypyruvic_acid_YID_00724	0.659617637	0.553964634
MONA_Malic acid	1.571352765	1.354224414
MASSBANK_L-(+)-Lysine; LC-ESI-QTOF; MS2; CE	0.998671604	0.86310566
MASSBANK_C10-LAS (TENTATIVE)	1.37761675	1.196172473
MASSBANK_Tryptophan; LC-ESI-ITFT; MS2; HCD; CE 90.0 eV; [M-H]-	0.958800503	0.837999872
METLIN_Xanthosine_MID_3408	1.618244312	1.43062595
RESPECT_5-AMINOVALERIC ACID	0.954678549	0.844339583

METLIN_Norethindrone_acetate_MID_44171	1.123186441	1.000423242
METLIN_Cystathionine_MID_63300	0.930820836	0.829832758
METLIN_D-Aspartic_acid_MID_63097	0.910156615	0.813465187
VGDB_Glycerophosphocholine_YID_01203	1.184078258	1.06217822
METLIN_Asn_Leu_Lys_MID_16424	0.822282657	0.738845957
METLIN_D-Erythrose_4-phosphate_MID_355	1.065082635	0.974279306
VGDB_1-Palmitoyl-2-hydroxy-sn-glycero-3-phosphoethanolamine_YID_11013	1.527709609	1.398667687
VGDB_Nicotinic_acid_adenine_dinucleotide_YID_11745	0.874621567	0.806254573
MONA_Asparagine	1.723145449	1.602722989
MONA_Dimethyl sulfoxide	1.549998782	1.454429539
VGDB_3-Methyl-L-histidine_YID_01435	0.879046551	0.827090583
METLIN_ADMA_MID_6891	1.103257918	1.04149395
MASSBANK_Cytidine 5'-diphosphocholine; LC-ESI-QTOF; MS2; CE	0.785581655	0.744231569
METLIN_PE(18	1.330648938	1.266440017
MASSBANK_Xanthosine; LC-ESI-QTOF; MS2; CE	1.593936595	1.552910998
VGDB_.beta.-Nicotinamide_adenine_dinucleotide_YID_00003	1.049633442	1.043550762
METLIN_5-L-Glutamyl-L-alanine_MID_58376	1.436071778	1.436872648
RESPECT_3-Hydroxy-3-Methylglutaric acid	0.865893825	0.868067898
METLIN_L-Glutamic_acid_MID_19	0.938256384	0.962093798
METLIN_Citric_acid_MID_124	0.970821386	0.996641282
METLIN_Pyridoxine_MID_2202	0.8354293	0.858488899
METLIN_N-Methylanthranilic_acid_MID_44613	0.824324906	0.84757245
MONA_Ornithine	0.997139132	1.025933462
VGDB_Cyclic_adenosine_diphosphate_ribose_YID_03552	1.051987312	1.083157939
METLIN_4-Nitrophenol_MID_4100	0.955531738	0.987576009
MASSBANK_Phthalic anhydride; LC-ESI-QTOF; MS2; [M+H]+; CE	1.247796308	1.290595143
MASSBANK_Adiphenine; LC-ESI-QTOF; MS2; CE	0.890037486	0.922276234
MASSBANK_Acetylcholine; LC-ESI-QTOF; MS2; CE	0.947984353	0.982712258
METLIN_L-Serine_MID_30	0.929171241	0.964372951
METLIN_Pro_Asp_MID_23776	0.969551948	1.010000567
MASSBANK_beta-Nicotinamide adenine dinucleotide; LC-ESI-QTOF; MS2; CE	0.977610767	1.022794298
MASSBANK_Citrulline; LC-ESI-QTOF; MS2; CE	0.933478193	0.984350745
MASSBANK_Niacinamide; LC-ESI-QTOF; MS2; CE	0.615502233	0.652636752
VGDB_Acetyl-DL-carnitine_YID_07777	0.863263728	0.916160807
MASSBANK_4-Hydroxybenzoylcholine; LC-ESI-QTOF; MS2; CE	0.860424734	0.914976857
MONA_Leucine	0.837896191	0.89175146
METLIN_Ile_Pro_Lys_MID_16048	1.026595871	1.102409944
VGDB_Galactonic_acid_YID_07692	0.558404993	0.599785228
VGDB_L-Methionine_YID_00595	0.879476057	0.946433752
METLIN_cis-Aconitic_acid_MID_3300	0.944837166	1.018172496

METLIN_Choline_MID_56	0.904820999	0.975628747
METLIN_3-Carboxypropyl_trimethylammonium_MID_34501	0.861809453	0.932878963
MONA_Tryptophan	0.887765951	0.969340194
VGDB_3,4,5-Trimethacarb_YID_07620	1.289564907	1.410661725
METLIN_DL-Phenylalanine_MID_65707	0.821547361	0.904328376
METLIN_L-Serine_MID_30	0.993733484	1.095055483
METLIN_D-(+)-Mannose_MID_136	1.159650815	1.279518846
METLIN_L-Phenylalanine_MID_28	0.924562364	1.024414656
VGDB_Betaine_YID_01224	0.890464341	0.991756971
VGDB_N-.alpha.-Acetyl-L-ornithine_YID_01020	0.876148757	0.977256386
METLIN_D-Pipecolic_acid_MID_58337	0.910227555	1.017625077
METLIN_D-Proline_MID_58150	0.798155247	0.8955686
VGDB_4-Hydroxyisophthalic_acid_YID_04460	0.846430125	0.965929306
MONA_Allantoin	0.808625877	0.922801253
MASSBANK_Glycerol-2-phosphate; LC-ESI-QTOF; MS2; CE	0.966732703	1.109725616
MASSBANK_Glutamic acid; LC-ESI-QTOF; MS2; CE	0.761286798	0.874146505
METLIN_L-Proline_MID_29	0.837358028	0.962139082
MASSBANK_L-Citrulline; LC-ESI-ITFT; MS2; m/z	0.821481197	0.962703851
VGDB_D-Aspartic_acid_YID_00987	0.714360485	0.840222231
METLIN_2-Pyrimidine_Acetic_Acid_MID_45029	0.860390785	1.013850099
METLIN_1-Aminocyclopropane-1-carboxylic_acid_MID_6029	0.787614691	0.935440793
MASSBANK_L-5-Oxoproline; LC-ESI-QTOF; MS2; CE	0.750906376	0.901167819
METLIN_5'-CMP_/_Cytidine-5-monophosphate_MID_3452	0.756604812	0.908971358
VGDB_L-Threonine_YID_00748	0.695360007	0.849223485
METLIN_D-Glutamine_MID_63630	0.854365373	1.050816078
VGDB_N-Fluorenylacetamide_YID_01872	0.837121498	1.030292429
MASSBANK_N-Acetyl-DL-aspartic acid; LC-ESI-QTOF; MS2; CE	0.896876274	1.124861834
RESPECT_N-Acetylneuraminate	0.986229576	1.239061314
METLIN_CMP-N-acetylneuraminic_acid_MID_434	0.853774357	1.078788083
RESPECT_Pantothenate	0.842207443	1.071959947
VGDB_L-Norvaline_YID_01642	0.910264617	1.171153325
VGDB_1-(1',3'-Benzodioxol-5'-yl)-2-butanamine_YID_07636	1.225617209	1.578178136
RESPECT_Pantothenate	0.821875467	1.059113282
METLIN_N-Acetyl-L-alanine_MID_5733	0.737603376	0.95258507
VGDB_Creatine_YID_00862	0.810436352	1.053234101
MONA_Abietic acid	0.512564861	0.666680777
MONA_N-Acetylneuraminic acid	0.951276727	1.247249185
MASSBANK_7,8-Didehydroastaxanthin; FAB-EBEB; MS2; m/z	0.661215242	0.869443374
RESPECT_S-Adenosyl-L-methionine	0.386505242	0.513894962
MONA_3-Hydroxyphenylalanine	0.844369946	1.129390861
METLIN_Trigonellinamide_MID_274	0.784813407	1.074917217

METLIN_GSH_/_Glutathione_MID_44	0.875627901	1.234249343
MASSBANK_C12-AS (TENTATIVE)	1.076645557	1.517782361
METLIN_Vinpocetine_MID_44335	0.697025095	0.988114348
METLIN_Phosphocholine_MID_3318	0.74477878	1.070484207
MONA_Glutathione (reduced)	0.700717273	1.014541084
METLIN_Quadrangolin_A;_2-[(1S,2S,4aR,8aS)-1-Hydroxy-4a-methyl-8-methylenedecahydro-2-naphthalenyl]acrylic_acid_MID_985308	0.897192879	1.303363558
METLIN_NADH_MID_3687	0.968746653	1.407726128
METLIN_C75_MID_44901	0.79314397	1.162645765
METLIN_Succinic_acid_MID_114	0.706768634	1.036175734
METLIN_DL-2-Aminoadipic_acid_MID_324	0.89521736	1.333610407
METLIN_D-Sorbitol_MID_143	0.59916624	0.9040978
MONA_N-Methylserine	0.700470269	1.063166814
VGDB_Indole-7-carboxaldehyde_YID_11618	0.748358307	1.148165821
VGDB_1-Oleoyl-2-hydroxy-sn-glycero-3-phospho-(1'-rac-glycerol)_YID_11182	0.865404942	1.334767205
MONA_Proline-hydroxyproline	0.756607263	1.181040054
MONA_4-Acetamidobutyric acid	0.678938397	1.060725527
MONA_Uridine-5-diphosphoacetylgalactosamine	0.680081364	1.066213634
VGDB_L-Carnosine_YID_00969	0.480360721	0.755944885
METLIN_2-Methylbutyroylcarnitine_MID_5367	0.793775179	1.253126909
METLIN_Myo-Inositol_MID_144	0.709902403	1.131500832
VGDB_(1R,5S)-8-Methyl-8-azabicyclo[3.2.1]octan-3-amine_YID_02035	0.518872626	0.828924064
METLIN_N-Acetylaspartate_MID_3769	0.702706109	1.126411749
VGDB_L-Propionylcarnitine_YID_07992	0.807486807	1.299397334
METLIN_D-Arabinono-1,4-lactone_MID_3272	0.723405261	1.202902846
METLIN_L-Glutamine_MID_18	0.786243449	1.328316767
METLIN_Uridine_diphosphate-N-acetylglucosamine_MID_41549	0.555753181	0.941081169
VGDB_Pyridoxine_YID_00873	0.586237819	0.99501286
VGDB_N-Glycolylneuraminic_acid_YID_01958	0.800204064	1.378275428
METLIN_UDPG_/_Uridine_5'-diphosphoglucose_MID_3598	0.529464096	0.916534355
METLIN_Ergothioneine_MID_53	0.508678063	0.884856277
METLIN_N1,N12-Diacetylspermine_MID_6525	0.567119367	1.013502728
MONA_Phthalic acid	0.900790926	1.633420021
METLIN_¦Á-D-Glucose_MID_133	0.502174816	0.911518823
METLIN_L-NMMA_MID_44311	0.680834976	1.241076868
METLIN_4-Hydroxybenzoic_acid_MID_3263	0.781713959	1.429113216
METLIN_5'-AMP_/_Adenosine_5'-monophosphate_MID_34478	0.488055944	0.917845823
VGDB_Creatine_phosphate_YID_01758	0.515880693	0.982563214
VGDB_D-Pyroglutamic_acid_YID_01742	0.739634326	1.411538774
METLIN_Aceclidine_MID_44245	0.898067109	1.718190089
METLIN_dGMP_/_2'-Deoxyguanosine_5'-monophosphate_MID_3489	0.477356265	0.9148082
VGDB_3-Furancarboxylic_acid,_tetrahydro-4-methylene-5-oxo-2-propyl-,_(2R,3S)-rel-_YID_07481	0.416102726	0.836608

METLIN_(¡À)-Propionylcarnitine_MID_85176	0.632943069	1.273776729
VGDB_Dulcitol_YID_01588	0.479617645	0.96862346
METLIN_O-Acetyl-L-serine_MID_3270	0.427058886	0.865459742
METLIN_Ala_His_MID_23724	0.510924404	1.036545664
METLIN_L-Carnitine_MID_52	0.469701339	0.953597352
VGDB_3-Deoxy-D-glycero-D-galacto-2-nonulosonic_acid_YID_08686	0.705320693	1.433473831
METLIN_Pro_Thr_Gln_MID_18145	0.684643284	1.398274895
METLIN_Uric_acid_MID_88	0.487707722	1.034840926
METLIN_Thr_Met_Lys_MID_17412	0.714665246	1.530497713
METLIN_(¡À)-Hexanoylcarnitine_MID_85174	0.482261981	1.037217903
METLIN_Butanoyl_PAF_MID_62940	0.528166803	1.14109501
METLIN_Gln_Leu_Lys_MID_23514	0.570374686	1.269673385
METLIN_L-Histidine_MID_21	0.152502761	0.348169163
METLIN_D-Glyceraldehyde_3-phosphate_MID_3294	0.473760675	1.083787506
VGDB_N-Acetyl-L-glutamic_acid_YID_01168	0.37446681	0.859631807
METLIN_N1-Acetylspermidine_MID_3323	0.438492899	1.032329745
VGDB_Tetraethylene_glycol_YID_07655	0.76746324	1.81031481
MONA_Cyclohexanamine	0.432030009	1.053338062
MASSBANK_Arginine; LC-ESI-ITFT; MS2; CE 80.0 eV; [M+H]+	0.372151295	0.921921308
VGDB_Sarsasapogenin_YID_02025	0.485837012	1.239520524
METLIN_Hypotaurine_MID_281	0.388709871	1.016579892
METLIN_Creatine_MID_7	0.375257732	1.002344521
METLIN_Sphinganine_MID_395	0.703795877	1.931228673
METLIN_N-Acetyl-L-glutamic_acid_MID_3325	0.333476649	0.922103376
VGDB_Erucamide_YID_04971	0.767299921	2.12322626
METLIN_Taurine_MID_31	0.301257765	0.860529948
METLIN_N-Acetylputrescine_MID_3252	0.914762359	2.652448062
MONA_Histidine	0.080044455	0.235067732
METLIN_5'-Methylthioadenosine_MID_3425	0.288281316	0.908460698
METLIN_N-Lactoyl-phenylalanine_MID_263655	0.329087834	1.112814229
MASSBANK_L-Histidine; LC-ESI-QTOF; MS2; CE	0.107256441	0.405758354
MASSBANK_L-(+)-Arginine; LC-ESI-QTOF; MS2; CE	0.28634095	1.086014328
VGDB_(R)-Butyrylcarnitine_YID_01888	0.315180744	1.351263132
VGDB_Spermidine_YID_00875	0.299278858	1.73012013
METLIN_Asp_Gln_Gly_MID_17811	0.476694722	4.171812588
METLIN_p-Tolyl_Sulfate_MID_263577	0.347986334	3.329659682
VGDB_(4-Aminobutyl)guanidine_YID_00732	0.492603996	5.312321641
VGDB_1-Methyl-L-histidine_YID_01434	0.158845992	2.079675252
METLIN_GSH_/_Glutathione_MID_44	0.147669944	1.938982085
VGDB_Pheophorbide_a_YID_03998	0.341278342	5.103853851
METLIN_Thioetheramide-PC_MID_63018	0.064297886	1.298216625
VGDB_Mono-2-ethylhexyl_phthalate_YID_01948	0.006396515	1.284909057

Table S2. Primer sequences for RT-qPCR in the research.

Gene	Species	Sequence(5′-3′)
MAPK1	human	F 5′-TACACCAACCTCTCGTACATCG-3′

		R 5′-CATGTCTGAAGCGCAGTAAGATT-3′
SGMS2	human	F 5′-CAAATTGCTATGCCCACTGAATC-3′<br>R 5′-GTTGTCAAGACGAGGTTGAAAAC-3
GNAI3	human	F 5′-AGCAATAGACGGTGCCTCAG-3′<br>R 5′-TGCGGTCGATCATCTTGCTT-3′
PPP2R5E	human	F 5′-TAGGTCTCAAGGCAAGCCTAT-3′<br>R 5′-CTGTCAAACAGCCTCTGCTTATT-3′
β-actin	human	F 5′-GGTCATCACTATTGGCAACG-3′<br>R 5′-ACGGATGTCAACGTCACACT-3′

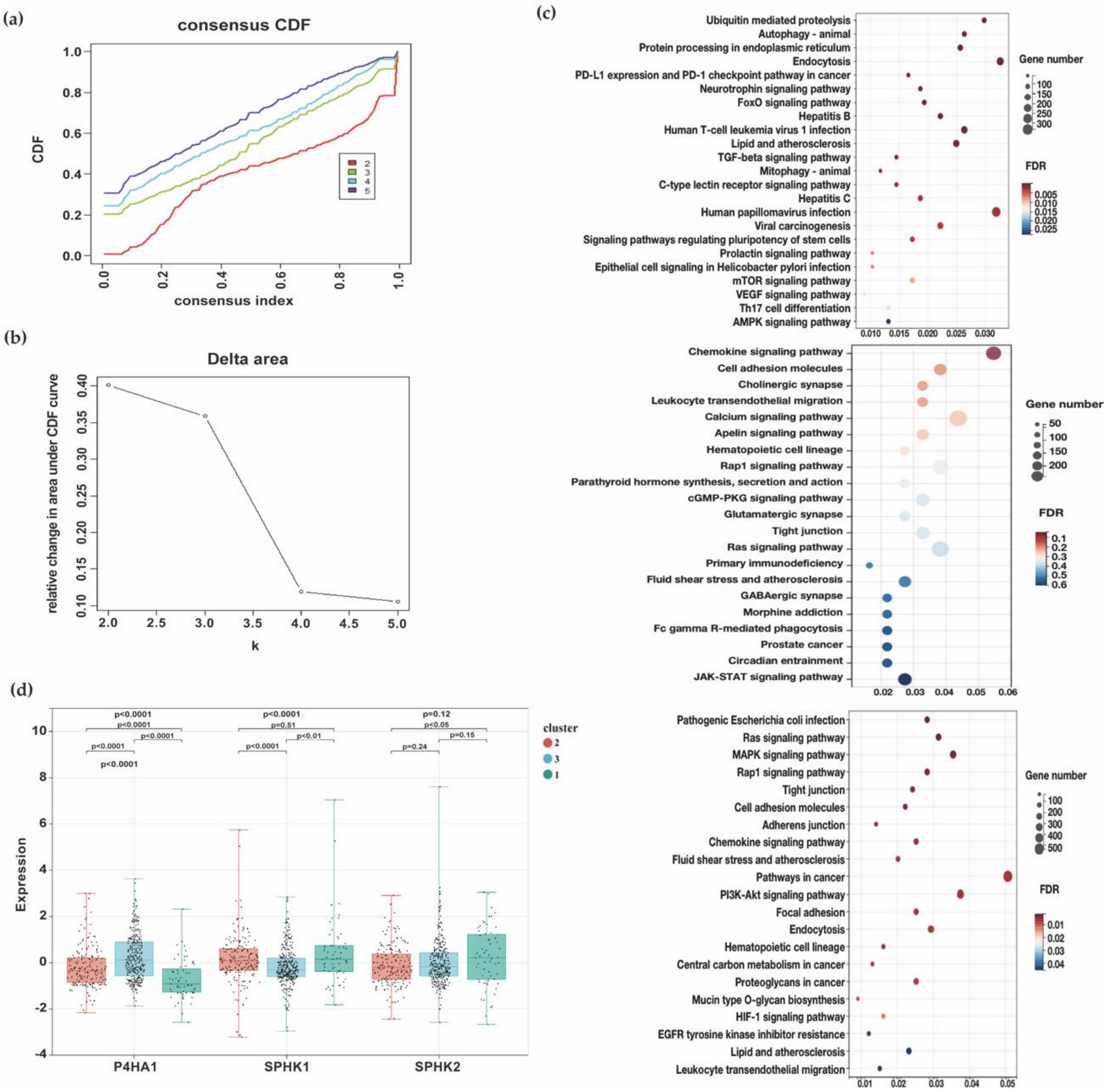

Figure S2. Extra information of PDAC subtypes (a). CDF curve of the merged dataset. (b). CDF delta area curve. (c). Functional enrichment analysis results for three subtypes. (d). Expression box plot of targeting genes in samples. (cluster1=59,cluster2=183,cluster3=321). (CDF:cumulative distribution function).

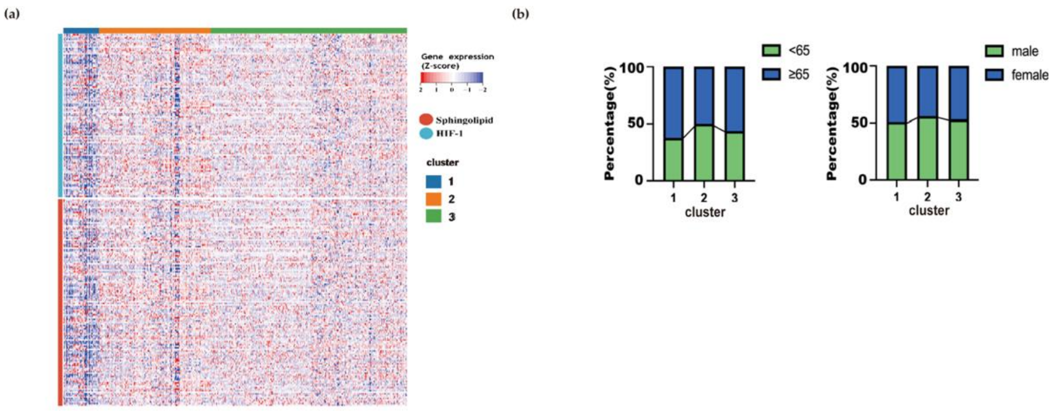

Figure S3. Distribution characters of clusters (cluster1=59,cluster2=183,cluster3=321) (a). The expression heatmap of cluster groups in hypoxia and sphingolipid metabolism pathways. (b).The distribution of clinical information about age and sex.
